# Research Status and Molecular Mechanism of the Traditional Chinese Medicine and Antitumor Therapy Combined Strategy Based on Tumor Microenvironment

**DOI:** 10.3389/fimmu.2020.609705

**Published:** 2021-01-21

**Authors:** Yang Zhang, Yanni Lou, Jingbin Wang, Cunguo Yu, Wenjuan Shen

**Affiliations:** ^1^ Department of Internal Medicine, First Affiliated Hospital, Heilongjiang University of Chinese Medicine, Harbin, China; ^2^ Oncology Department of Integrated Traditional Chinese and Western Medicine, China-Japan Friendship Hospital, Beijing, China; ^3^ Department of Spleen and Stomach Disease, Chinese Medicine Shenzhen Hospital, Guangzhou University, Shenzhen, China; ^4^ Department of Chinese Medicine, Qinhuangdao Haigang Hospital, Qinhuangdao, China; ^5^ Department of Obstetrics and Gynecology, First Affiliated Hospital, Heilongjiang University of Chinese Medicine, Harbin, China

**Keywords:** traditional Chinese medicine, cancer, immune ability, anti-tumor therapy, tumor microenvironment

## Abstract

Treatment of malignant tumors encompasses multidisciplinary comprehensive diagnosis and treatment and reasonable combination and arrangement of multidisciplinary treatment, which is not a simple superimposition of multiple treatment methods, but a comprehensive consideration of the characteristics and specific conditions of the patients and the tumor. The mechanism of tumor elimination by restoring the body’s immune ability is consistent with the concept of “nourishing positive accumulation and eliminating cancer by itself” in traditional Chinese medicine (TCM). The formation and dynamic changes in the tumor microenvironment (TME) involve many different types of cells and multiple signaling pathways. Those changes are similar to the multitarget and bidirectional regulation of immunity by TCM. Discussing the relationship and mutual influence of TCM and antitumor therapy on the TME is a current research hotspot. TCM has been applied in the treatment of more than 70% of cancer patients in China. Data have shown that TCM can significantly enhance the sensitivity to chemotherapeutic drugs, enhance tumor-suppressing effects, and significantly improve cancer-related fatigue, bone marrow suppression, and other adverse reactions. TCM treatments include the application of Chinese medicine monomers, extracts, classic traditional compound prescriptions, listed compound drugs, self-made compound prescriptions, as well as acupuncture and moxibustion. Studies have shown that the TCM functional mechanism related to the positive regulation of cytotoxic T cells, natural killer cells, dendritic cells, and interleukin-12, while negatively regulating of regulatory T cells, tumor-associated macrophages, myeloid-derived suppressive cells, PD-1/PD-L1, and other immune regulatory factors. However, the application of TCM in cancer therapy needs further study and confirmation. This article summarizes the existing research on the molecular mechanism of TCM regulation of the TME and provides a theoretical basis for further screening of the predominant population. Moreover, it predicts the effects of the combination of TCM and antitumor therapy and proposes further developments in clinical practice to optimize the combined strategy.

## Introduction

Cancer is one of the major noncommunicable chronic diseases that seriously affect human health (1–3). Although treatment methods and drug research and development continue to improve, many problems such as drug resistance and recurrence still hinder progress (4–9). The treatment of malignant tumors encompasses multidisciplinary comprehensive diagnosis and treatment (10–12) and reasonable combination and arrangement of multiple treatment methods including surgery, radiotherapy, chemotherapy, targeted therapy, immunotherapy, endocrine therapy, interventional therapy (13–19). Multidisciplinary treatment is not a simple superimposition of multiple treatment methods, but a comprehensive consideration of the characteristics and specific conditions of the patients and the tumor, leading to a planned and reasonable choice (20). While pursuing prolonged survival (21), attention should also be given to improving the quality of life of the patients (22).

Due to its huge population, China accounts for about a quarter of the world’s new tumors and deaths, leading to a serious disease burden (23–25). Traditional Chinese medicine (TCM) is a unique diagnosis and treatment method with thousands of years of history (26, 27). It is reported that most Chinese cancer patients have used TCM during the diagnosis and treatment process (28, 29). TCM is mostly used in the form of compound prescriptions in clinical oncology, including oral herbal medicines, granules or capsules, and injections (30). There are many pieces of research on Chinese medicine monomers and their active ingredients (31). Many researches have shown that TCM combined with antitumor therapy can achieve significant tumor suppression effects, reduce drug resistance, and improve adverse reactions and patient quality of life (32–36). In recent years, targeting the immune checkpoints CTLA-4, PD-1, and PD-L1 has led to breakthroughs in a variety of cancer types (37–39). The mechanism of tumor eradication by restoring the body’s immune ability is consistent with the concept of “nourishing positive accumulation and eliminating cancer by itself” or “strengthening vital Qi to treat cancer” in TCM (40, 41).

*The tumor microenvironment (TME)* (42, 43) is formed by the structural components such as tumor cells, endothelial cells, fibroblasts, immune cells, extracellular matrix, and secreted cytokines. It has three main roles: inhibiting the immune response, promoting angiogenesis, and growing cancer stem cells. Chronic inflammation (44, 45) and immunosuppression (46, 47) are the core features of the TME. Chronic inflammation leads to low oxygen levels, low pH, high pressure in the microenvironment, and the prolonged existence of inflammatory factors such as tumor necrosis factor (TNF) that maintain and continuously aggravate the inflammatory features of the TME. The hypoxic microenvironment increases hypoxia inducible factor (HIF) levels, induces the formation of new blood vessels, modifies the vascular endothelial growth factor (VEGF), and recruits bone marrow-derived endothelial progenitor cells to form new blood vessels. The TME enables a large number of regulatory T cells (Tregs) that penetrate and accumulate in tumor tissues, inhibit the differentiation and maturation of effector cells such as lymphocytes, macrophages, dendritic cells (DC), and isolate them from tumor tissues to inhibit immune responses. The immunosuppressive microenvironment is closely related to the “deficiency of vital Qi” in Chinese medicine (48). The “syndrome” of TCM involves multiple systems and levels of Western medicine. TCM treatment of cancer pays attention to overall regulation whether it is to strengthen the body (Fu Zheng) or eliminate evil (Qu Xie). Its advantage lies in regulating the tumor-host microenvironment, allowing normal immune cells to perform their duties, so that there is no environment for tumor cells to survive, and causing apoptosis or autophagy (49–51).

In this review, we mainly discuss, from the perspective of TME regulation, the studies on the combined application of TCM and anticancer treatments. Further screening of dominant populations and predictors will help optimize the joint strategy and provide a theoretical basis for clinical practice.

## TCM Combined With Chemotherapy

Chemotherapy is still the cornerstone of anticancer therapy. As one of the most important treatments for advanced stage cancer, chemotherapy compatibility ensures the correct combination of chemotherapeutic drugs and the combination of chemotherapy and other types of treatment. There are many clinical and preclinical studies on the combination of TCM treatment and chemotherapy.

The clinical studies on TCM combined with chemotherapy for anticancer treatment research have been mainly published in Chinese journals (**Table 1**). The studied cancer types include lung cancer (52–58), digestive system cancer (gastric/liver/esophageal cancer) (59–63), gynecological cancer (ovarian cancer, choriocarcinoma, breast cancer) (64–68), and bladder cancer (69). The observed drugs are mainly compound herbal medicines, including classic prescriptions [Baihe Gujin decoction (52), Yanghe decoction (61)], listed drugs [Shenqi Fuzheng injection (57, 64, 67), Kanglaite injection (58), compound Kushen injection (59), Aidi injection (59), Jinfukang oral liquid (54), Yifei Qinghua granules (55)], a variety of self-made empirical formulas (53, 56, 60, 62, 63, 65, 66), and monomeric Chinese medicines or their components (matrine) (68, 69). The results consistently show that TCM can help improve the sensitivity to chemotherapeutic drugs, enhance tumor-suppressing effects, and significantly improve cancer-related fatigue, bone marrow suppression, and other adverse reactions. The quality of life self-report scores also show significant improvement. Regarding the regulation of tumor immune function, clinical studies mainly detected immune-related factors in peripheral blood. The results concluded that TCM combined with chemotherapy can upregulate CD3^+^, CD4^+^, and CD4^+^/CD8^+^ (52, 55, 59, 60, 64, 65, 67, 68), interleukin-2 (IL-2) (52, 62, 66), interferon-gamma (INF-γ) (52, 61, 66, 68), natural killer cells (NK) (55, 69), and cytotoxic T lymphocytes (CTL) (69), while downregulating IL-6 (55, 59, 66, 68), IL-10 (52, 61), transforming growth factor-β1 (TGF-β1) (59, 61), vascular endothelial growth factor(VEGF) (58, 62), matrix metalloproteinase-2 (MMP-2), MMP-9 (54, 56, 62), Forkhead box protein 3 (Foxp3), and B7-H3 (53), and Tregs (57, 61, 69). However, there are also inconsistencies between different research results for some indicators.

**Table 1:** Influence of traditional Chinese medicine (TCM) Combined Chemotherapy (CT) on tumor microenvironment (TME)-Clinical Study.

Cancers	Formulation	Herbal medicine	Group and Number of cases	Effect of Combination	Upregulate	Downregulate	Refs.
Lung Cancer							
NSCLC	Compound (Classic, oral)	Baihe Gujin Decoction (Rehmannia, Rehmannia, Carlo, album AGLAOPHOTIS, lilium, Fritillaria, Ophiopogon, Bellflower, Scrophulariaceae, rudis Licoricia)	TCM + DC vs DC = 48 vs 48	DCR 89.6% vs 72.9% (p<0.05)	CD3^+^, CD4^+^, CD4^+^/CD8^+^, IL-2, and INF-γ	CD8^+^, IL-4, IL-10	He and Huang (52)
NSCLC	Compound (Self-made, oral)	Bushen Yifei Jiedu Decoction (Xianling lienis contegentem membranam, Ligustrum lucidum, Ganoderma lucidum, Cruda Astragalus, Radix Angelicae Ginseng, Pinellia, Atractylodes, Germen Stone, aesculus aesculus, Shansi Fungorum aurea Buckwheat, etc)	TCM + CT vs CT = 30 vs 30	/	/	Foxp3, B7-H3	Wang et al. (53)
NSCLC	Compound (Listed drug, Oral Liquid)	Jinfukang Oral Liquid (Astragalus, Ophiopogon japonicus, Adenophora glabra, Ligustrum lucidum, Cornus alba, etc)	TCM + G vs G = 60 vs 60	DCR 91.67% vs 75% (p<0.05)	/	MMP-9	Zhang et al. (54)
NSCLC	Compound (Listed drug, oral)	Yifei Qinghua Granule (American Ginseng, Adenophora, Astragalus, Ophiopogon japonicus, Patrinia vulgaris, Oldenlandia diffusa, Panax notoginseng, etc)	TCM + CT vs CT = 101 vs 101	DCR 81.19% vs 64.36% (p<0.05)	CD3^+^, CD4^+^, CD4^+^/CD8^+^, NK	CD8^+^, IL-6, TNF-α, hs-CPR	Wang et al. (55)
NSCLC	Compound (Self-made, oral)	Yifei Xiaoai Decoction (Houttuynia, Cattus BRACHIUM, Mountain Conch, Codonopsis, Asparagus, Fritillaria, Populus tremula Reed Stein, Loquat Folium, Panax notoginseng, Citrus aurantium, STILIO, etc)	TCM + TP vs TP = 32 vs 32	DCR 84.4% vs 68.9% (p<0.05)	/	VEGF, MMP-2, MMP-9	Liu and Yang (56)
NSCLC	Compound (Listed drug, injection)	Shenqi Fuzheng Injection (Codonopsis, Astragalus)	TCM + TP vs TP = 41 vs 38	DCR 90.2% vs 76.3%, PFS 19m vs 13m, OS 43m vs 29m (p<0.05)	Th17	Treg	Chen et al. (57)
NSCLC	Compound (Listed drug, injection)	Kanglaite Injection (Coix Oil)	TCM + GP vs GP = 36 vs 36	DCR 91.67% vs 69.34% (p<0.05)	/	VEGF, P53, anti-Survivin antibody	Wang et al. (58)
Digestive system cancer							
Gastric Cancer	Compound (Listed drug, injection)	Compound Kushen Injection (Sophora flavescens, Berberis vulgaris uniseriale) Aidi Injection (Astragalus, Acanthopanax senticosus, Ginseng, Cantharidin)	TCM + FOLFOX vs FOLFOX = 39 vs 39	DCR 97.44% vs 87.18% (p<0.05)	hydrogen sulfide, CD3^+^, CD4^+^, CD8^+^, CD4^+^/CD8^+^	IL-6, TGF-β1	Meng et al. (59)
Gastric Cancer	Compound (Self-made, oral)	Buzhong Guben Yiwei Decoction (Atractylodes movent-frixum, Ginseng, Astragalus, Poria, tangerine cortices, Chuanxiong, Carlo, Fritillaria, Cyperus rotundus, album AGLAOPHOTIS, Bupleurum falcatum, amomi, Platycodon, lignei, Licoricia, gingiberi, Jujube)	TCM + FOLFOX vs FOLFOX = 50 vs 50	DCR 66% vs 38% (p<0.05)	CD3^+^, CD4^+^, CD4^+^/CD8^+^	/	Wang and Zhang (60)
Gastric Cancer	Compound (Classic, oral)	Yanghe Decoction (Muta Rehmannia, cornibus gum, eruca alba semina, ephedra, cinnamo, gingiberi, Licoricia)	TCM + DOX vs DOX = 60 vs 60	DCR 85% vs 68.3% (p<0.05)	IFN-γ	HIF-1α, IL-10, TGF-β1, TNF-α, MCP-1, MDSCs, Tregs	Tian et al. (61)
Hepatocelllar Carcinoma	Compound (Self-made, oral)	Chaihu Zaoxiu Decoction (Bupleurum falcatum, Fusarium oxysporum, Poria, rubrum AGLAOPHOTIS, album AGLAOPHOTIS, Rubia, Angelica, Turmeric, Cyperus rotundus: Scutellaria, Curcuma, totum cucumis, Cruda turtur, Polygonum cuspidatum, Licorice)	TCM + FAP vs FAP = 40 vs 40	ORR 35% vs 22.5% (p<0.05)	IL-2	MMP-2, MMP-9, VEGF, IGF-2	Wang and Jiao (62)
Esophageal Cancer	Compound (Self-made, oral)	Buyi Zhiai Decoction (Ginseng, Poria, Astragalus, Carlo, album AGLAOPHOTIS, Rehmannia, Atractylodes, Licoricia, Shouwu, CISSANTHEMOS, dandelion, Taraxacum)	TCM + DDP vs DDP = 53 vs 53	/	CD4^+^, CD4^+^/CD8^+^, IgA, IgG, IgM	CD8^+^	Feng et al. (63)
Gynecological cancer							
Ovarian Cancer	Compound (Listed drug, injection)	Shenqi Fuzheng Injection (Codonopsis, Astragalus)	TCM + TC vs TC = 55 vs 50	DCR 70.9% vs 50% (p<0.05)	CD3^+^, CD4^+^, CD4^+^/CD8^+^	/	Wei and Li (64)
Choriocarcinoma	Compound (Self-made, oral)	Fuzheng Yiliu Formula (Astragalus, Ligustrum lucidum, Oldenlandia diffusa, Scutellaria barbata, Fritillaria, Salvia, muta Rehmannia, Codonopsis, rubrum AGLAOPHOTIS, futurist awards, Poria, Atractylodes, Carlo, Polygonatum, Zihe, Gynostemma, Pinellia, Licoricia, aurantiaco cortices, three-lens)	TCM + EMA-CO vs EMA-CO = 46 vs 46	DCR 73.91% vs 47.83% (p<0.05)	CD3^+^, CD4^+^, CD4^+^/CD8^+^	CD8^+^	Zhang et al. (65)
Triple-negative Breast Cancer	Compound (Self-made, oral)	Huangqi Jiedu Decoction (Astragalus, Scutellaria barbata, Coix semen Oldenlandia diffusa, Radix Angelicae Scrophulariaceae, Gentianaceae, Ligustrum lucidum, solanum, Atractylodes, Poria Cocos, Ophiopogon japonicus: Fungorum Shanzi, Fritillaria, Licorice)	TCM + GT vs GT = 51 vs 51	DCR 78.4% vs 58.8% (p<0.05)	IL-2, IFN-γ, IL-2/IL-6, IFN-γ/IL-6	IL-6	Yang et al. (66)
Breast Cancer	Compound (Listed drug, injection)	Shenqi Fuzheng Injection (Codonopsis, Astragalus)	TCM + D vs DEC = 50 vs 50	DCR 84% vs 64% (p<0.05)	CD3^+^, CD4^+^, CD4^+^/CD8^+^, CD80, CD83, CD86	CD8^+^	Lu et al. (67)
Cervical Cancer	Monomer (Listed drug, injection)	Matrine Injection	TCM + DC vs DC = 35 vs 35	DCR 94.29% vs 71.43% (p<0.05)	IL-18, IFN-γ, CD3^+^, CD4^+^, CD4^+^/CD8^+^	IL-6	Du et al. (68)
Others							
Bladder Cancer	Monomer (Listed drug, injection)	Matrine Injection	TCM + GP vs GP = 39 vs 42	DCR 97.43% vs 87.5% (p = 0.034), PFS 12.64m vs 10.37m, OS 18.63m vs 15.09m (p<0.001)	CTL, NK	Treg	Han et al. (69)

Experimental studies were conducted through in vivo and in vitro research (**Table 2**). Cell and animal experiments present more in-depth research on the mechanism of TCM improvement of chemotherapeutic efficacy. The observed drugs include Chinese medicine monomers [curcumin (70–73), ginsenoside Rg3 (74)], extracts [Ginseng and Astragalus (75)], classic traditional compound prescriptions [Huangqin decoction PHY906 (76, 77), Shiquan Dabu decoction (78)], listed compound drugs [Shexiang Baoxin pill (79)], and self-made compound prescriptions (80, 81). The most representative ones are curcumin, PHY906, and tonic Chinese medicines. The combination of curcumin and chemotherapy has been proven to overcome multidrug resistance [FOLFOX (70), oxaliplatin (71, 73), 5-Fu (72)] through a number of in vivo and in vitro studies. The effect of this combined therapy may upregulate Bax, caspase-3, and PARP and downregulate EGFRs (such as IGF-1R), Bcl‐2, survivin, HSP70, Nrf2, Bcl-2/Bax, NF-κB, p-p65, and TGF-β/Smad2/3. PHY906 is derived from the classic formula prescription Huangqin decoction; however, instead of the separation and purification of the possible active compounds, it is taken as a whole. In-depth research via animal experiments, clinical trials, and quality control of PHY906 have been conducted. Results showed that PHY906 could significantly increase the antitumor activity of CPT-11, decreasing toxicity in normal tissues while promoting cell death within the TME, and that its effect may be upregulated by IRF-1, IRF-5, CCL-2/MCP-1, and CCL-5/RANTES. Tonic Chinese medicines include monomers, water extracts, the classic compound Shiquan Dabu decoction, or self-made prescriptions, and their mechanism of action may be related to the regulation of macrophage polarization, the reduction of epithelial cell-mesenchymal transition, and cell stemness.

**Table 2:** Influence of traditional Chinese medicine (TCM) Combined Chemotherapy (CT) on tumor microenvironment (TME) - Experimental Study.

Cells	Type	Formulation	Herbal medicine	Chemo	Effect of Combination	Upregulate	Downregulate	Refs.
HCT-116 and HT-29; LoVo-xenograft; HCT−8/5−Fu cells	In vitro and in vivo	Monomer	Curcumin	FOLFOX/Oxa/5−Fu	Confirmed the reversal effect on multidrug resistance	Bax, caspase-3, and PARP	EGFRs and IGF-1R; Bcl‐2, survivin, HSP70; Nrf2, Bcl-2/Bax; NF-κB; p-p65, TGF-β/Smad2/3	Patel et al. (70) Guo et al. (71) Zhang et al. (72) Yin et al. (73)
SPC-A1, H1299, A549 cell lines	In vitro and in vivo	Monomer	Ginsenoside Rg3	DDP	Increase the sensitivity to DDP	/	NF-κB, EMT and stemness	Wang et al. (74)
A549 cells and Lewis lung carcinoma (LLC1)	In vitro in and vivo	Compound extract	WEGA (Water extract of ginseng and astragalus)	DDP	Effectively inhibited the transplanted tumor growth and improved weight loss and immunosuppressive	inhibited A549 cell proliferation, M1 macrophage markers	M2 TAMs markers	Chen et al. (75)
Colon 38 tumors	In vivo	Compound (Classic)	PHY906 (Glycyrrhiza uralensis Fisch, Paeonia lactiflora Pall, Scutelleriabaicalensis Georgi, and Ziziphus jujuba Mill )	CPT-11	Increased the antitumor activity of CPT-11 while decreasing animal weight loss caused by CPT-11; decrease toxicity in normal tissues while promoting cell death within the tumor microenvironment	IRF-1, IRF-5, CCL-2/MCP-1 and CCL-5/ RANTES	/	Lam et al. (76) Wang et al. (77)
B6F10 Melanoma cells	In vitro and in vivo	Compound (Classic)	Shiquan Dabu Decoction (Ginseng, calami similiter ducentos quinquaginta, Chuanxiong, Rehmannia, Poria, Atractylodes, Licoricia, Astragalus, Carlo, etc)	DDP	Inhibit the proliferation of melanoma, promote apoptosis and the proliferation of mouse spleen lymphocytes, enhance the anti-tumor effect	IL-4	IL-1β	Zhang et al. (78)
Murine LLC cells	In vitro and in vivo	Compound (Listed drug)	Shexiang Baoxin Pill (Artificial Moschus, Cortex cinnamomic, Borneolum, Radix ginseng, Calculus bovis, Styrax, Venenum Bufonis)	GEM	Enhance the effective treatment performancewhile minimizing the toxic side effects	tumor angiogenesis, blood perfusion, vascular permeability, and vessel dilation	a-SMA, collagen	Yang et al. (79)
Lewis lung carcinoma (LLC1)	In vitro and vivo	Compound (Self-made)	**JC-001** (Bupleurum chinense DC, Gentiana scabra Bge., Rheum palmatum L., Clematis montana Buch.-Ham., Carthamus tinctorius L., Prunus persica (L.) Batsch , Angelicadahurica (Fisch. ex Hoffm.) Benth. et Hook. f., Siegesbeckia orientalis L., Glycyrrhiza uralensis Fisch. and Solanum incanum L.)	DDP	JC-001 suppressed foci formation and reduced the viability of LLC1 cells in vitro, increased the chemosensitivity to CDDP in vivo	IL-2, IL-10, TNF-α and INF-γ, enhanced the Th1 response	IL-17A, TGF-β	Chuang et al. (80)
CNE2 cells nasopharyngeal carcinoma	In vivo	Compound Granules	**Qi-Boosting Toxin-Resolving Granules** (Rudis Astragalus, Coptis, Oldenlandia diffusa, etc)	DDP	Apoptotic rate was significantly higher	INF-γ, Foxp3	IL-17, TGF-β, ROR-γt mRNA	Hu et al. (81)

## TCM Combined With Targeted Therapy or Immunotherapy

Unlike the destructive antitumor effects of traditional chemotherapy, new molecular targeted therapies target specific molecular changes in cancer (16). They have achieved significant effects in clinical practice in recent years and have also triggered a change in the concept of anticancer treatment. The immunotherapeutic approach involves the restart and maintenance of the tumor immune cycle, the restoration of the body**’**s normal antitumor immune response, and the control and elimination of cancer, by means such as monoclonal antibody immune checkpoint inhibitors, therapeutic antibodies, tumor vaccines, cell therapy, and small molecule inhibitors. Among them, PD-1 inhibitors lead the treatment of malignant tumors into a new era of immunotherapy (17, 38, 39). There are currently many studies on the combined application of TCM and targeted drugs or immunotherapy, focusing on improving efficacy, reversing drug resistance, and reducing adverse reactions. Some research results describing the impact of TCM combined with targeted drugs (**Table 3**) and immunotherapy (**Table 4**) on the TME have been released; however, there are still more treatment aspects that need further clarification.

**Table 3:** Influence of TCM Combined Targeted therapy.

Cancers	Type	Formulation	Herbal medicine	Targeted therapy	Effect of Combination	Upregulate	Downregulate	Refs.
NSCLC	Clinical	Compound (Classic, oral)	Xuefu Zhuyu Decoction (Angelica, peach kernel, chuanxiong, safflower, red peony root, achyranthes, bupleurum, citrus aurantium, platycodon, habitat, mountain mushroom, oldenlandia diffusa, shuyangquan, gecko, licorice)	TCM + gefitinib/erlotinib vs gefitinib/erlotinib = 39 vs 39	DCR 56.4% vs 48.7% (p<0.05)	T cell, Th, Tc	Protease activated receptor (PAR)-1 expression in tumor tissue	Liet al. (82)
Hepatocellular Carcinoma	Clinical	Compound (Listed drug, injection)	Shenqi Fuzheng Injection (Codonopsis, Astragalus)	TCM + sorafenib vs sorafenib = 44 vs 44	DCR 97.7% vs 86.4% (p<0.05)	CD4^+^	CD8^+^, CXCR3, miR-103	Lu et al. (83)
Ovarian cancer	Clinical	Compound (Self-made, oral)	Yiqi Yangyin Decoction (Seres yam, Astragalus, Habitat, Polygonatum, Scrophulariaceae, Ligustrum lucidum, Zingiber turmeric, Shanzi fungus Prunella vulgaris, Platycodon grandiflorum, Jujube)	TCM + bevacizumab vs bevacizumab = 43 vs 37	/	CD3^+^, CD4^+^, CD4^+^/CD8^+^, NK IL-2, INF-γ	CD4^+^CD25^+^, IL-6, IL-10, VEGF, CD133, DDX4	Guli et al. (84)
HCC cells	in vitro and in vivo	Compound (Listed drug, injection)	Compound Kushen Injection (Sophora flavescens, Berberis vulgaris uniseriale)	sorafenib	Enhanced the anticancer activity of sorafenib at a subclinical dose with no obvious side effects.	triggering TNFR1, mediated NF-κB and p38 MAPK signaling cascades	/	Yang et al. (85)
Gastric AGS cells	in vitro	Monomer component	Astragalus polysaccharide (APS)	apatinib	Remarkable increase in apoptosis	/	p-AKT, MMP-9	Wu et al. (86)
Pancreatic cancer cell lines	in vitro	Monomer component	Astragalus polysaccharide (APS)	apatinib	Enhanced inhibitory effects on cell migration and invasion, and increased cell apoptosis percentage	/	p-AKT, p-ERK, MMP-9	Wu et al. (87)
HepG2 xenografts	in vivo	Compound (Classic, oral)	PHY906 (KD018)	sorafenib	potentiate the anti-hepatoma activity	hMCP1, M1/M2, AMPKα-P and ULK1-S555-P, ERK1/2-P	/	Lam et al. (88)

**Table 4:** Influence of traditional Chinese medicine (TCM) Combined Immunotherapy.

Cancers	Type	Formulation	Herbal medicine	Immunotherapy	Effect of Combination	Upregulate	Downregulate	Refs.
WEHI-3 leukemia or EL4 lymphoblast cells	in vitro	Monomer component	PG2 (A polysaccharide isolated from the radix of Astragalus membranaceus)	/	*via* the protein kinase B (Akt)/mammalian target of rapamycin (mTOR)/ribosomal protein S6 kinase beta-1 (p70S6K) pathway	/	PD-L1 on the cell surface	Chang et al. (91)
Diffuse large B cell lymphoma	in vitro	Monomer component	Ginsenoside Rg3	PD-1 inhibitor	enhances the anti-tumor effect and proliferation of T cells, inhibiting apoptosis, increasing the secretion of cytokine	IL-2, IFN-γ	/	Guo et al. (92)
TC-1	in vivo	Monomer	Glycyrrhiza uralensis water extract	HPV dendritic cell-based vaccine	dose-dependently promoted DC maturation and cytokine secretion through TLR4 signaling pathway	CD4+ and CD8+ T cells	iTregs: CD4+CD25-Fopx3+	Aipire et al. (93)
MB79 bladder cancer	in vivo	Monomer component	Bisdemethoxycurcumin	α-PD-L1	significantly prolonged survival of intraperitoneal metastasized bladder cancer bearing mice	intratumoral CD8^+^ T-cell infiltration, IFN-γ in the blood	intratumoral MDSCs	Shao et al. (94)
CT26 colon cancer	in vivo	Compound (Classic)	Gegen Qinlian Decoction	anti-mouse PD-1	Gut microbiota analysis revealed that combination significantly enriched for s: Bacteroides_acidifaciens and s:uncultured_organism_g:norank_f:Bacteroidales_S24-7_group.	CD8+ T cells in peripheral blood, IFN-γ, IL-2,	PD-1	Lv et al. (95)
CT26 colon cancer	in vivo	Compound (Classic)	ninjin'yoeito (NYT, Renshen Yangrong Decoction)	tumor vaccine	synergistically enhances the effects of the prophylactic tumor vaccine	CD8+ T cells	Tregs: CD4+CD25-Fopx3+	Takaku et al. (96)
B16 melanoma cancer	in vivo	Compound (Classic)	Juzentaihoto (JTT, Shiquan Dabu Decoction)	anti-PD-1 antibody	significantly suppressed B16 cell metastasis	IL-12, IFN-γ, NK	/	Ishikawa et al. (97)
Advanced stage cancer	Clinical	Compound (Classic, oral)	Guipi Decoction	NK cell	TCM symptoms improved (shortness of breath, over fatigued, tiredness)	CD3^+^, CD4^+^, CD8^+^, NK	/	Lu and Peng (98)
Castration-resistant prostate cancer	Clinical	Compound (Classic, oral)	Hochu-ekki-to (Buzhong Yiqi Decoction) and Keishi-bukuryo-gan (Guizhi Fuling Pills)	Personalized Peptide Vaccine (PPV)	well tolerated without severe adverse events.		Mo-MDSC, IL-6	Koga et al. (99)

In addition to evaluating the immune function of peripheral blood, some studies involved the detection of tumor-specific markers and tumor tissue-related factors to evaluate the invasion ability of tumors. Clinical studies on non-small-cell lung carcinoma (NSCLC) (82), hepatocellular carcinoma (83), and ovarian cancer (84) use compound preparations, including classic traditional formula Xuefu Zhuyu decoction (82) and listed compound herbal medicine Shenqi Fuzheng injection (83). During *in vitro* and *in vivo* studies, apart from observing the effects on tumor cell proliferation, the related signaling pathways were explored, including the NF-κB and p38 MAPK signaling cascades mediated by TNFR1 in hepatocellular carcinoma cells (compound Kushen injection) (85) and the AKT pathway in gastric cancer and pancreatic cancer cells (Astragalus polysaccharide) (86, 87). YIV-906, which is based on PHY906 (88), is a clinical observation drug that ensures **>**90% consistency in product quality (89). It is also the first Chinese medicinal project to be awarded a grant from the PO1 program of the National Cancer Center of the United States. Animal experiments show that PHY906 may potentiate sorafenib action and that its mechanism of action involves an increase in hMCP1 expression, enhanced infiltration of macrophages into tumors with a higher M1/M2 expression pattern, and upregulation of AMPKα-P and ULK1-S555-P. Computer simulation methods have also been used in the analysis of its mechanism of action and key components (90).

Whether tonic herbal medicine be used in combination with immunotherapy is one of the issues that Chinese cancer patients are extremely concerned about; moreover, it is a very controversial issue for cancer clinicians. Research on the combination of TCM and immunotherapy mainly includes ***in vivo*** and ***in vitro*** studies, while clinical studies are rarely conducted. The TCM studied mostly include tonic drugs or their components: Astragalus (91), ginsenoside Rg3 (92), Glycyrrhiza uralensis water extract (93), and bisdemethoxycurcumin (94). Most of the compound prescriptions are classic medicines, including Gegen Qinlian decoction (95), Renshen Yangrong decoction (96), Shiquan Dabu decoction (97), Guipi decoction (98), and Buzhong Yiqi decoction (99). The components of Astragalus can downregulate PD-L1 on the tumor cell surface, which may be related to the AKT/mTOR/p70S6K pathway (91). *In vivo* studies have shown that TCM combinations have a positive effect on therapeutic curative potential and tumor inhibition. Some studies also explored the intestinal flora; however, in clinical observation, the main observed effect remains the improvement of symptoms. Both TCM treatment and immunotherapy have systematic and complex characteristics. Determining whether TCM affects the efficacy or the adverse effects of immunotherapy by regulating the TME and related factors necessitates further research.

## TCM Combined With Local Treatment

Malignant tumors require different treatment strategies according to the different stages of the disease. Additionally, local treatment plays an important role in the treatment of cancer. Early radical surgery is the most effective way to obtain a curative effect and long-term survival. Radiotherapy and interventional therapy can obtain survival benefits and symptom improvement through the control of local lesions. The combined citation of TCM and local treatment have been clinically observed to reduce perioperative complications, promote the recovery of immune function, reduce recurrence and metastasis, and improve long-term prognosis. The TCM involved are mostly listed drugs (**Table 5**), and research on their mechanism of action is relatively lacking and limited to peripheral blood immune function detection.

**Table 5:** Influence of traditional Chinese medicine (TCM) Combined Local treatment (Peri-operation, γ-knife, interventional therapy).

Cancer	Type	Formulation	Herbal medicine	Local treatment	Effect of Combination	Upregulate	Downregulate	Refs.
NSCLC	Clinical	Compound (Listed drug, injection)	Aidi Injection	Neoadjuvant Chemo: TCM + Neo-Chemo vs Neo-Chemo = 35 vs 20	Decreased blood loss in operation: 530. 15 ± 104. 55 ml vs 615. 49 ± 114. 08 ml (P<0.05); Increased curative effect: 45. 71% vs 30. 00% (P<0.05); Decreased leucopenia: 37. 14% vs 60. 00% (P<0.05)	/	CD4^+^, CD25^+^	Teng et al. (100)
Lung cancer	Clinical	Compound (Listed drug, oral)	Zilongjin Tablets	Post operation: TCM vs control = 30 vs 30	promote the recovery of immune function	IgA, IgG, CD4^+^, CD4^+^/CD8^+^, NK	/	Huang et al. (101)
Triple-negative Breast Cancer	Clinical	Compound (Self-made, oral)	Shenghe Powder	Post operation Chemo-Radio Therapy(CRT): TCM + CRT vs CRT = 84 vs 84	5-year survival rate: 76. 2% vs 64. 3% (P<0.05); Recurrence and metastasis rate: 25.0% vs 39.3% (P<0.01)	CD3^+^, CD4^+^, CD4^+^/CD8^+^, NK; miR-34a,	LDHA	Wang et al. (102)
Primary liver cancer	Clinical	Compound (Listed drug, injection)	Compound Danshen Injection	TCM + γ-knife vs γ-knife = 20 vs 20	/	IgA, IgG, IgM, CD3^+^, CD4^+^, CD4^+^/CD8^+^,	/	Zhu and Yi (103)
Primary liver cancer	Clinical	Compound (Listed drug, oral)	Jinlong Capsule	TCM + intervention vs intervention = 30 vs 30	facilitates the cellular immunity recovery	keep stable: CD3^+^, CD4^+^, CD4^+^/CD8^+^, NK; SIL-2R, TSGF	/	Yuan et al. (104)

## Discussion

The clinical application of TCM has a long history, and its treatment principles and philosophy have a unique system. With the continuous improvement of research methods, our understanding of TCM is deepening. The study of herbal medicine monomers and their components is relatively easy to explain; however, compound prescription and compatibility are more characteristic of TCM holistic thinking. TCM has its advantages and specifics in the treatment of cancer. In addition to reducing the side effects of antitumor treatment and improving the symptoms and patient quality of life, it also **“**supports the healthy Qi**”** and restores the body**’**s own immune system. It can improve efficacy and prolong survival in the comprehensive treatment of cancer.

Many studies on the monomers or components of herbal medicine have confirmed that they affect related factors in the TME; however, their effects in a more complex system are relatively unexplored. This review summarizes and analyzes the influence and effect of TCM in combination with antitumor therapy, including chemotherapy, targeted therapy, immunotherapy, the perioperative period, radiotherapy, and interventional therapy. Relevant Chinese medicines include marketed drugs (injections, oral liquids, and tablets), traditional prescriptions, and self-developed experiential prescriptions, as well as many Chinese medicinal monomers or ingredients. Tonic drugs are the main active agents, including multiple treatments such as replenishing Qi, invigorating the spleen, promoting blood circulation, eliminating phlegm, clearing heat, and dispelling stagnation. It is well known that the immune system of body, plays defensive, protective and eliminative roles on tumor cells. For example, NK cells can directly recognize and eradicate tumor cells; Dendritic cells (DCs) can activate adaptive immunity; macrophages (M) can kill tumor cells by generating cytotoxicity, which related to the production of effector molecules and accompanying phagocytosis. Clinical studies have shown that adding TCM to the treatment strategy can significantly improve patient symptoms without increasing adverse reactions, with a tendency to prolong survival. The detection of peripheral blood-related immune factors suggests that TCM has a regulatory effect on immune function and that it can promote a healthy Th1/Th2 balance and regulate the polarization of macrophages. Peripheral blood is the most commonly used medium for disease diagnosis and has been widely accepted by patients for noninvasive molecular diagnosis. In addition, compared with the tumor tissue sample, the dynamic change of microenvironment is ignored, and the peripheral blood can be sampled for many times regularly, which is convenient for monitoring. In relevant *in vivo* and *in vitro* studies, possible mechanisms of action have been discussed, including the classical NF-κB, AKT, and TLR4 signaling pathways and the intestinal flora. However, TCM treatment still needs to go through top-level design, good quality control, reverse verification, and in-depth research that can reproduce results to demonstrate the role of TCM in the comprehensive treatment of tumors and clarify its therapeutic mechanism.

## Conclusions

Cancer treatment has multiple stages and high complexity, and the optimal approach includes multidisciplinary comprehensive diagnosis and treatment. TCM has its unique advantages and characteristics that are different from other types of antineoplastic treatment, and these should not be ignored. However, current research results cannot clearly explain the dominant population and mechanism of effect of TCM combined with antitumor therapy; however, the impact on the TME may be the core principle of this approach. More evidence-based experimental research is still needed to provide a basis for formulating better combined strategies for cancer treatment.

## Author Contributions

YZ, YL, and WS concepted and designed the review. YZ and YL wrote the manuscript. JW, CY, and WS revised the manuscript. All authors contributed to the article and approved the submitted version.

## Funding

This work is supported by the National Natural Science Foundation of China (No. 81973601 and No. 81904003), and the Beijing Municipal Natural Science Foundation (No. 7202184).

## Conflict of Interest

The authors declare that the research was conducted in the absence of any commercial or financial relationships that could be construed as a potential conflict of interest.

## Abbreviations

Non-small cell lung cancer, NSCLC; Chemotherapy, CT; Traditional Chinese medicine, TCM; Disease control rate, DCR; Interleukin 2, IL-2; Interleukin 10, IL-10; Interferon-*γ*, IFN-*γ*; Gemcitabine, G; Matrix metalloprotein-2, MMP-2; Matrix metalloprotein-9, MMP-9; Natural killer cells, NK; Tumor necrosis factor α, TNF-α; High sensitivity C-reactive protein, hs-CRP; Vascular endothelial growth factor, VEGF; Gemcitabine & cisplatin, GP; Paclitaxel & cisplatin, TP; Transforming growth factor β 1, TGF-β_1_; Fluorouracil & Oxaliplatin & calcium folinate, FOLFOX; Doxorubin, DOX; Hypoxia inducible factor-1 α, HIF-1α; Monocyte chemoattractant protein-1, MCP-1,; Myeloid suppressor cells, MDSCs; Insulin-like growth factor-2, IGF-2; Fluorouracil & adriamycin & chloramphenicol, FAP; Cisplatin, DDP; Paclitaxel & cyclophosphamide, TC; Methotrexate & actinomycete D & calcium folinate & vincristine sulfate & cyclophosphamide, EMA-CO; Paclitaxel & gemcitabine, GT; Cisplatin & docetaxel, DC; Gemcitabine & Cisplatin, GP.

## References

[1] Freddie BBMP JF ME Isabelle Soerjomataram MD MP, MPH RLS, MSPH LAT, Ahmedin Jemal PhD D. Global cancer statistics 2018: GLOBOCAN estimates of incidence and mortality worldwide for 36 cancers in 185 countries. CA: A Cancer J Clin (2018) 68:394–424. 10.3322/caac.21492 30207593

[2] Fidler MM Bray F Soerjomataram I The global cancer burden and human development: A review. Scand J Public HEALT (2018) 46:27–36. 10.1177/1403494817715400 28669281

[3] Camilla M Giuseppe L Cancer statistics: a comparison between World Health Organization (WHO) and Global Burden of Disease (GBD). Eur J Public Health (2019) 30:1026–27. 10.1093/eurpub/ckz216 31764976

[4] Neil V José B M HD A view on drug resistance in cancer. Nature (2019) 575:299–309. 10.1038/s41586-019-1730-1 31723286 PMC8008476

[5] Nikolaou M Pavlopoulou A Georgakilas AG Kyrodimos E The challenge of drug resistance in cancer treatment: a current overview. Clin Exp Metastas (2018) 35:309–18. 10.1007/s10585-018-9903-0 29799080

[6] Ankur G Kumardeep C Rahul K Sudheer G Harinder S RGP S Managing Drug Resistance in Cancer: Role of Cancer Informatics. Methods Mol Biol (Clifton NJ) (2016) 1395:299–312. 10.1007/978-1-4939-3347-1_17 26910081

[7] Assaraf YG Brozovic A Gonçalves AC Jurkovicova D Linē A Machuqueiro M The multi-factorial nature of clinical multidrug resistance in cancer. Drug Resist Update (2019) 46:100645. 10.1016/j.drup.2019.100645 31585396

[8] M GM Orit L D HM Jean-Pierre G Toward a Better Understanding of the Complexity of Cancer Drug Resistance. Annu Rev Pharmacol (2016) 56:85–102. 10.1146/annurev-pharmtox-010715-103111 26514196

[9] N AS Robert E R SE D SCM Fear of cancer recurrence: A qualitative systematic review and meta-synthesis of patients’ experiences. Clin Psychol Rev (2019) 68:13–24. 10.1016/j.cpr.2018.12.001 30617013

[10] Pillay B Wootten AC Crowe H Corcoran N Tran B Bowden P The impact of multidisciplinary team meetings on patient assessment, management and outcomes in oncology settings: A systematic review of the literature. Cancer Treat Rev (2016) 42:56–72. 10.1016/j.ctrv.2015.11.007 26643552

[11] Rogers MJ Matheson L Garrard B Maher B Cowdery S Luo W Comparison of outcomes for cancer patients discussed and not discussed at a multidisciplinary meeting. Public Health (2017) 149:74–80. 10.1016/j.puhe.2017.04.022 28575751

[12] PhD IPD MB MPH CA MPH Kathleen M Castro RN MA Brandys B multidisciplinary treatment planning in US cancer care settings. Outcomes Cancer-Am Cancer Soc (2018) 124:3656–67. 10.1002/cncr.31394 30216477

[13] Wyld L Audisio RA Poston GJ The evolution of cancer surgery and future perspectives. Nat Rev Clin Oncol (2015) 12:115–24. 10.1038/nrclinonc.2014.191 25384943

[14] Schaue D McBride WH Opportunities and challenges of radiotherapy for treating cancer. Nat Rev Clin Oncol (2015) 12:527–40. 10.1038/nrclinonc.2015.120 PMC8396062 26122185

[15] Stefan B Molecular precision chemotherapy: overcoming resistance to targeted therapies? Clin Cancer Res (2014) 20:1064–6. 10.1158/1078-0432.CCR-13-3194 24536061

[16] Lee YT Tan YJ Oon CE Molecular targeted therapy: Treating cancer with specificity. Eur J Pharmacol (2018) 834:28–32. 10.1016/j.ejphar.2018.07.034 30031797

[17] Guo Q Huang F Goncalves C Rincón SVD Miller WH Translation of cancer immunotherapy from the bench to the bedside. Adv Cancer Res (2019) 143:1–62. 10.1016/bs.acr.2019.03.001 31202357

[18] Fabian CJ Visvanathan K Somerfield MR Use of Endocrine Therapy for Breast Cancer Risk Reduction: ASCO Clinical Practice Guideline Update Summary. J Oncol Pract (2019) 15:607–10. 10.1200/JOP.19.00379 31479306

[19] AS S CM R TP T KP G BG M Helen B Clinical Development of Novel Drug-Radiotherapy Combinations. Clin Cancer Res An Off J Am Assoc Cancer Res (2019) 25:1455–61. 10.1158/1078-0432.CCR-18-2466 PMC6397668 30498095

[20] Linda H Cath T Magda Z Ross W Emma P James G Improving the effectiveness of cancer multidisciplinary team meetings: analysis of a national survey of MDT members’ opinions about streamlining patient discussions. BMJ Open Qual (2019) 8:e000631. 10.1136/bmjoq-2019-000631 PMC6567952 31259288

[21] Stone E Rankin N Kerr S Fong K Currow DC Phillips J Does presentation at multidisciplinary team meetings improve lung cancer survival? Findings from a consecutive cohort study. Lung Cancer (2018) 124:199–204. 10.1016/j.lungcan.2018.07.032 30268461

[22] Elena CM Romano D Corrado G Pietro I Alessandra E Massimo U Management of toxicities associated with targeted therapies for HR-positive metastatic breast cancer: a multidisciplinary approach is the key to success. Breast Cancer Res TR (2019) 176:483–94. 10.1007/s10549-019-05261-5 PMC6586706 31065872

[23] Wu C Li M Meng H Liu Y Niu W Zhou Y Analysis of status and countermeasures of cancer incidence and mortality in China. Sci China(Life Sci) (2019) 62:640–7. 10.1007/s11427-018-9461-5 30900169

[24] Feng RM Zong YN Cao SM Xu RH Current cancer situation in China: good or bad news from the 2018 Global Cancer Statistics? Cancer Commun (2019) 39:22. 10.1186/s40880-019-0368-6 PMC6487510 31030667

[25] Wanqing Chen PhD MD RZ MPH PDB P BMedSc SZ Hongmei Zeng PhD MD PhD FB Cancer statistics in China, 2015. CA: A Cancer J Clin (2016) 66:115–32. 10.3322/caac.21338 26808342

[26] Ma Y Zhou K Fan J Sun S Traditional Chinese medicine: potential approaches from modern dynamical complexity theories. Front Med-PRC (2016) 10:28–32. 10.1007/s11684-016-0434-2 26809465

[27] Xu Q Bauer R Hendry BM Fan TP Zhao Z Duez P The quest for modernisation of traditional Chinese medicine. BMC Complem Altern M (2013) 13:132. 10.1186/1472-6882-13-132 PMC3689083 23763836

[28] So T Chan S Lee V Chen B Kong F Lao L Chinese Medicine in Cancer Treatment - How is it Practiced in the East and the West? Clin ONCOL-UK (2019) 31:578–88. 10.1016/j.clon.2019.05.016 31178347

[29] Nie J Zhao C Deng L Chen J Yu B Wu X Efficacy of traditional Chinese medicine in treating cancer (Review). Biomed Rep (2016) 4:3–14. 10.3892/br.2015.537 26870326 PMC4726876

[30] Liu J Mao JJ Wang XS Lin H Evaluation of Traditional Chinese Medicine Herbs in Oncology Clinical Trials. Cancer J (2019) 25:367–71. 10.1097/PPO.0000000000000404 31567465

[31] Xiang Y Guo Z Zhu P Chen J Huang Y Traditional Chinese medicine as a cancer treatment: Modern perspectives of ancient but advanced science. Cancer Med (2019) 8:1958–75. 10.1002/cam4.2108 PMC6536969 30945475

[32] Liu X Li M Wang X Dang Z Yu L Wang X Effects of adjuvant traditional Chinese medicine therapy on long-term survival in patients with hepatocellular carcinoma. Phytomedicine (2019) 62:152930. 10.1016/j.phymed.2019.152930 31128485

[33] Tang M Wang S Zhao B Wang W Zhu Y Hu L Traditional Chinese Medicine Proton vs Progression-Free Survival and Enhances Therapeutic Effects in Epidermal Growth Factor Receptor Tyrosine Kinase Inhibitor (EGFR-TKI) Treated Non-Small-Cell Lung Cancer (NSCLC) Patients Harboring EGFR Mutations. Med Sci Monitor (2019) 25:8430–7. 10.12659/MSM.917251 PMC6865232 31704907

[34] Huang S Peng W Mao D Zhang S Xu P Yi P Kangai Injection, a Traditional Chinese Medicine, Improves Efficacy and Reduces Toxicity of Chemotherapy in Advanced Colorectal Cancer Patients: A Systematic Review and Meta-Analysis. Evid-Based Compl Alt (2019) 2019:8423037. 10.1155/2019/8423037 PMC6662435 31379968

[35] Wong W Chen BZ Lee AKY Chan AHC Wu JCY Lin Z Chinese Herbal Medicine Effectively Prolongs the Overall Survival of Pancreatic Cancer Patients: A Case Series. Integr Cancer Ther (2019) 18:1534735419828836. 10.1177/1534735419828836 30791742 PMC6432679

[36] FuuJen T Xiang L ChaoJung C TeMao L JianShiun C PoHeng C Chinese herbal medicine therapy and the risk of overall mortality for patients with liver cancer who underwent surgical resection in Taiwan. Complement Ther Med (2019) 47:102213. 10.1016/j.ctim.2019.102213 31780007

[37] Muenst S Läubli H Soysal SD Zippelius A Tzankov A Hoeller S The immune system and cancer evasion strategies: therapeutic concepts. J Intern Med (2016) 279:541–62. 10.1111/joim.12470 26748421

[38] Hongxing S Shih-Hsin YE Marty C John F Carlo C BJ A Predictive biomarkers for immune checkpoint blockade and opportunities for combination therapies. Genes Dis (2019) 6:232–46. 10.1016/j.gendis.2019.06.006 PMC6997608 32042863

[39] Andrews LP Yano H Vignali DAA Inhibitory receptors and ligands beyond PD-1, PD-L1 and CTLA-4: breakthroughs or backups. Nat Immunol (2019) 20:1425–34. 10.1038/s41590-019-0512-0 31611702

[40] Jinjing W Fanghua Q Zhixue W Zhikun Z Ni P Lei H A review of traditional Chinese medicine for treatment of glioblastoma. Biosci Trends (2020) 13:476–87. 10.5582/bst.2019.01323 31866614

[41] Tian JH Xi ZC Luo B Que ZJ Xu HX Liu JX Scientific Connotation of the Theory of “Strengthening Vital Qi to Treat Cancer”. Modernization Tradit Chin Med Mater Med-World Sci Technol (2019) 21:943–8. 10.11842/wst.2019.05.017

[42] HD C SL A The Tumor Microenvironment Innately Modulates Cancer Progression. Cancer Res (2019) 79:4557–66. 10.1158/0008-5472.CAN-18-3962 PMC6744958 31350295

[43] Pitt JM Marabelle A Eggermont A Soria JC Kroemer G Zitvogel L Targeting the tumor microenvironment: removing obstruction to anticancer immune responses and immunotherapy. Ann Oncol (2016) 27:1482–92. 10.1093/annonc/mdw168 27069014

[44] IN M Ayca G DA J HC A Obesity and Cancer Mechanisms: Tumor Microenvironment and Inflammation. J Clin Oncol (2016) 34:4270–6. 10.1200/JCO.2016.67.4283 PMC5562428 27903155

[45] Glauben L De la Fuente Peti T Chanitra T HM A Chronic inflammation and cytokines in the tumor microenvironment. J Immunol Res (2014) 2014:149–85. 10.1155/2014/149185 PMC4036716 24901008

[46] Dingzhi W DR N Immunosuppression associated with chronic inflammation in the tumor microenvironment. Carcinogenesis (2015) 36:1085–93. 10.1093/carcin/bgv123 PMC5006153 26354776

[47] Nakamura K Yaguchi T Ohmura G Kobayashi A Kawamura N Iwata T Involvement of local renin-angiotensin system in immunosuppression of tumor microenvironment. Cancer Sci (2018) 109:54–64. 10.1111/cas.13423 29034589 PMC5765296

[48] Du J Cheng BCY Fu XQ Su T Li T Guo H In vitro assays suggest Shenqi Fuzheng Injection has the potential to alter melanoma immune microenvironment. J Ethnopharmacol (2016) 194:15–9. 10.1016/j.jep.2016.08.038 27566207

[49] Zhaokun Y Zijun L Jiumao L Anticancer Properties of Traditional Chinese Medicine. COMB Chem High T SCR (2017) 20:423–9. 10.2174/1386207320666170116141818 28093974

[50] Lin W Lu J Cheng B Ling C Progress in research on the effects of traditional Chinese medicine on the tumor microenvironment. J Integr Med (2017) 15:282–7. 10.1016/S2095-4964(17)60345-5 28659232

[51] Qiujun G Jie L Hongsheng L Effect and Molecular Mechanisms of Traditional Chinese Medicine on Regulating Tumor Immunosuppressive Microenvironment. BioMed Res Int (2015) 2015:261620. 10.1155/2015/261620 26161392 PMC4486742

[52] He SM Huang QQ Effect of Baihe Gujin Decoction with TP chemotherapy on advanced non-small cell lung carcinoma differentiated by lung-kidney yin deficiency and on the immune function. World J Integrated Tradit Western Med (2020) 15:682–686+690. 10.13935/j.cnki.sjzx.200425

[53] Wang JY Lin Y Li HG Zhou L Zhu LH Zhao LH Effect of Bushen Yifei Jiedu decoction combined with chemotherapy on expression of serum Foxp3 and B7-H3 in advanced non-small cell lung cancer of type of lung and kidney deficiency. Modern J Integrated Tradit Chin Western Med (2020) 29:1608–11. 10.3969/j.issn.1008-8849.2020.15.003

[54] Zhang JH Wang J Cui QR Effect of Jinfukang Oral Liquid Combined with Gemcitabine on Serum Level of MMP-9 in the Treatment of Non-Small Cell Lung Cancer with Deficiency of Both Qi and Yin. Inf Tradit Chin Med (2019) 36:65–8. 10.19656/j.cnki.1002-2406.190048

[55] Wang ZF Geng L Wang S Liu XB Zhang RX Liu SL Effect of Feiliuping ointment combined with chemotherapy on cellular immune function and related inflammatory factors in patients with lung cancer. Chin Tradit Herbal Drugs (2018) 49:5368–72. 10.7501/j.issn.0253-2670.2018.22.022

[56] Liu JX Yang LL Yifei Xiaoai Decoction in Treatment of Advanced Non-small Cell Lung Cancer. Acta Chin Med (2018) 33:2064–8. 10.16368/j.issn.1674-8999.2018.11.489

[57] Chen R Li M Ji BY Qi SJ Clinical Observation of Shenqi Fuzheng Injection Combined with Chemotherapy in Treatment of Non Small Cell Lung Cancer and The Regulation of Th17/Treg Cells in Patients. Chin Arch Tradit Chin Med (2018) 36:1994–7. 10.13193/j.issn.1673-7717.2018.08.054

[58] Wang Y Hui S Li M Zhang CH Clinical trial of Kanglaite injection in gemcitabine combined cisplatin regimen chemotherapy for advanced non-small cell lung cancer. Chin J Clin Pharmacol (2017) 33:2354–2356+2360. 10.13699/j.cnki.1001-6821.2017.23.008

[59] Meng XY Meng WY Ye Q Dai L Hu J Wang XH Effect of Modified FOLFOX7 Regimen Combined With Compound Kushen Injection and Aidi Injection on Levels of CA125,CEA,IL-6,TGFβ1 and Hydrogen Sulfide in Advanced Gastric Cancer and Analysis of Drug Safety. Chin Arch Tradit Chin Med (2020) 38:208–11. 10.13193/j.issn.1673-7717.2020.07.048

[60] Wang DD Zhang K Clinical study of Buzhong Guben Yiwei decoction combined with chemotherapy in the treatment of patients with advanced gastric cancer after operation. Hebei J Tradit Chin Med (2020) 42:240–244+248. 10.3969/j.issn.1002-2619.2020.02.018

[61] Tian TD Yang F Yue LY Wang XX Shi B Effect of Yanghetang in Chemotherapeutic Enhancement Response Among Patients of Advanced Gastric Cancer with Syndrome of Yang Deficiency and Cancer Inflammatory Factors Treg and MDSCs. Chin J Exp Tradit Med Formulae (2016) 22:160–4. 10.13422/j.cnki.syfjx.2016220160

[62] Wang KR Jiao HJ Clinical Efficacy of Chaihu Zaoxiu Decoction Combined with Chemotherapy in Treatment of Primary Hepatocellular Carcinoma and Its Influence on Related Serum Indicators. Chin Arch Tradit Chin Med (2020) 38:173–6. 10.13193/j.issn.1673-7717

[63] Feng XF Effect of Buyi Zhiai Decoction on Quality of Life and Immune Function in Patients with Middle and Late Stage Esophageal Cancer Chemotherapy. Chin Arch Tradit Chin Med (2019) 37:1006–8. 10.13193/j.issn.1673-7717.2019.04.057

[64] Wei HL Li LN The Influence of the Life Quality of Shengqi Fuzheng Injection on the Immune Function of Ovarian Cancer after Chemotherapy. Pract J Cancer (2019) 34:1032–4. 10.3969/j.issn.1001-5930.2019.06.046

[65] Zhang BP Meng M Lv SY Therapeutic effects on choriocarcinoma treated with fuzheng yiliu formula and EMA-CO chemotherapy and the impacts on immune function in the patients. World J Integrated Tradit Western Med (2019) 14:86–89+93. 10.13935/j.cnki.sjzx.190122

[66] Yang SY Gao ZQ Liu LL Lai Q Xie D Curative Effects of Huangqi Jiedu Decoction Combined with GT Chemotherapy on Metastatic Triple-negative Breast Cancer and its Influence on Th1/Th2 Cytokine Balance. Anti-tumor Pharm (2019) 9:312–316+324. 10.3969/j.issn.2095-1264.2019.02.29

[67] Lu R Shen ZW Cao LY Effect of Shenqi Fuzheng Injection combined with Docetaxel on Cellular Immune Function in Patients with Terminal Breast Cancer. Chin J Surg Integrated Tradit Western Med (2018) 24:149–55. 10.3969/j.issn.1007-6948.2018.02.005

[68] Du R Zhang YY Hu QF Effect of Matrine Injection on Chemosensitivity, Quality of Life and Immune Function in Patients with Cervical Cancer. Chin Arch Tradit Chin Med (2018) 36:3051–5. 10.13193/j.issn.1673-7717.2018.12.059

[69] Han ZX Zhao YQ Liu QJ Ji SQ Cheng WL Zhang HJ Short-term Effects of Matrine Injection Combined with Gemcitabine + Cisplatin Chemotherapy on Advanced Bladder Cancer Patients and on the Survival Time. Anti-tumor Pharm (2019) 9:112–6. 10.3969/j.issn.2095-1264.2019.01.24

[70] PB B Radha S Sadia Q HEtal V Yingjie Y RA K Curcumin enhances the effects of 5-fluorouracil and oxaliplatin in mediating growth inhibition of colon cancer cells by modulating EGFR and IGF-1R. Int J Cancer (2008) 122:267–73. 10.1002/ijc.23097 17918158

[71] Li-da G Yong-qing S Xiao-han Z Li-jian G Zhi-jun Y Duo W Curcumin combined with oxaliplatin effectively suppress colorectal carcinoma in vivo through inducing apoptosis. Phytother Res PTR (2015) 29:357–65. 10.1002/ptr.5257 25418925

[72] Chao Z Lian-Jun H Hai-Zhu Y Ding-Feng L Yi-Bao Z Dong-Dong M Nrf2 is a key factor in the reversal effect of curcumin on multidrug resistance in the HCT−8/5−Fu human colorectal cancer cell line. Mol Med Rep (2018) 18:5409–16. 10.3892/mmr.2018.9589 PMC6236280 30365132

[73] Jiahuan Y Li W Yong W Hailong S Xiaojie W Lei W Curcumin reverses oxaliplatin resistance in human colorectal cancer via regulation of TGF-β/Smad2/3 signaling pathway. Oncotarg Ther (2019) 12:3893–903. 10.2147/OTT.S199601 PMC6529728 31190888

[74] Wang J Tian L Khan MN Zhang L Liu J Ginsenoside Rg3 sensitizes hypoxic lung cancer cells to cisplatin via blocking of NF-κB mediated epithelial–mesenchymal transition and stemness. Cancer Lett (2018) 415:73–85. 10.1016/j.canlet.2017.11.037 29199005

[75] Yingna C Lei B Huijuan L Yucui J Feiyan C Yunshan W Water extract of ginseng and astragalus regulates macrophage polarization and synergistically enhances DDP’s anticancer effect. J Ethnopharmacol (2018) 232:11–20. 10.1016/j.jep.2018.12.003 30529424

[76] Lam W Bussom S Guan F Jiang Z Zhang W Gullen EA The Four-Herb Chinese Medicine PHY906 Reduces Chemotherapy-Induced Gastrointestinal Toxicity. Sci Transl Med (2010) 2:45ra59. 10.1126/scitranslmed.3001270 20720216

[77] Wang E Bussom S Chen J Quinn C Bedognetti D Lam W Interaction of a traditional Chinese Medicine (PHY906) and CPT-11 on the inflammatory process in the tumor microenvironment. BMC Med Genomics (2011) 4:38. 10.1186/1755-8794-4-38 21569348 PMC3117677

[78] Zhang TT Zou W Yang CM Qian C Wu YY Li XM Study on the Anti-Melanoma Effect of Shiquan Dabu Decoction and its Combination Therapy with Cisplatin. J Nanjing Univ Tradit Chin Med (2019) 35:160–5. 10.14148/j.issn.1672-0482.2019.0160

[79] Yang LQ Li RY Yang XY Cui QF Zhang JG Co-administration of Shexiang Baoxin Pill and Chemotherapy Drugs Potentiated Cancer Therapy by Vascular-Promoting Strategy. Front Pharmacol (2019) 10:565. 10.3389/fphar.2019.00565 31178734 PMC6543272

[80] Chuang M Jan M Chang JT Lu F The Chinese medicine JC-001 enhances the chemosensitivity of Lewis lung tumors to cisplatin by modulating the immune response. BMC Complem Altern M (2017) 17:210. 10.1186/s12906-017-1728-x PMC5387375 28399860

[81] Hu M Zhou FL Song L Li L He YC Effect of Qi-Boosting Toxin-Resolving Granules and cisplatin on transplanted tumor of nasopharyngeal carcinoma and balance of Foxp3/ROR-γt. Chin Tradit Herbal Drugs (2019) 50:2121–6. 10.7501/j.issn.0253-2670.2019.09.016

[82] Li Y Jiang LF Ma DY Cui QL Hu YH Fu BF Effect and Mechanism of Resolving Stasis and Detoxication Method Combined with EGFR-TKI on NSCLC Based on Modulating PAR1 to Remodel Immune Surveillance. Liaoning J Tradit Chin Med (2020) 47:145–7+222. 10.13192/j.issn.1000-1719.2020.06.044

[83] Lu XY Hua GD Bai J Tian XL Liu C Li YH Effect of Shenqi Fuzheng injection combined with sorafenib on miR-103,CXCR3 and AFP in postoperative patients with liver cancer. Chin J Integrated Tradit Western Med Liver Dis (2020) 30:218–20+230+ 290. 10.3969/j.issn.1005-0264.2020.03.009

[84] Gulimire· BRJ Reziya· KEB Ailikemu· ABDWK Li XW Gulina· KEB Effects of Yiqi Yangyin decoction combined with bevacizumab on immune function, inflammatory factors and tumor markers in patients with ovarian cancer. Oncol Prog (2019) 17:827–30. 10.11877/j.issn.1672-1535.2019.17.07.23

[85] Yang Y Mayu S Wenbo Y Feng W Xiaoguang L Wei W Compound kushen injection relieves tumor-associated macrophage-mediated immunosuppression through TNFR1 and sensitizes hepatocellular carcinoma to sorafenib. J Immunother Cancer (2020) 8:e000317. 10.1136/jitc-2019-000317 32179631 PMC7073790

[86] Jun W Junxian Y Jing W Chenguang Z Kun S Xiaojun Y Astragalus polysaccharide enhanced antitumor effects of Apatinib in gastric cancer AGS cells by inhibiting AKT signalling pathway. Biomed Pharmacother Biomed Pharmacother (2018) 100:176–83. 10.1016/j.biopha.2018.01.140 29428665

[87] Jun W Jing W Qiang S Wei D Teng L Junxian Y Traditional Chinese medicine Astragalus polysaccharide enhanced antitumor effects of the angiogenesis inhibitor apatinib in pancreatic cancer cells on proliferation, invasiveness, and apoptosis. Oncotarg Ther (2018) 11:2685–98. 10.2147/OTT.S157129 PMC5953274 29785118

[88] Wing L Zaoli J Fulan G Xiu H Rong H Jing W PHY906(KD018), an adjuvant based on a 1800-year-old Chinese medicine, enhanced the anti-tumor activity of Sorafenib by changing the tumor microenvironment. Sci REP-UK (2015) 5:9384. 10.1038/srep09384 PMC4377583 25819872

[89] Chang H Kuo Y Wu L Chang C Cheng K Tyan Y The extracts of Astragalus membranaceus overcome tumor immune tolerance by inhibition of tumor programmed cell death protein ligand-1 expression. Int J Med Sci (2020) 17:939–45. 10.7150/ijms.42978 PMC7163360 32308547

[90] Guo YW Guo XC Zhang JB Liu AC Quan LN Effect of Ginsenoside Rg3 Enhancing the Immunity of PD-1 Inhibitor in Treatment of DLBCL: A Study in Vitro. Acta Chin Med Pharmacol (2018) 46:24–9. 10.19664/j.cnki.1002-2392.180140

[91] Aipire A Li J Yuan P He J Hu Y Liu L Glycyrrhiza uralensis water extract enhances dendritic cell maturation and antitumor efficacy of HPV dendritic cell- based vaccine. Sci Rep-UK (2017) 7. 10.1038/srep43796 PMC5341557 28272545

[92] Shao Y Zhu W Da J Xu M Wang Y Zhou J Bisdemethoxycurcumin in combination with alpha-PD-L1 antibody boosts immune response against bladder cancer. Oncotarg Ther (2017) 10:2675–83. 10.2147/OTT.S130653 PMC5449128 28579805

[93] Lv J Jia Y Li J Kuai W Li Y Guo F Gegen Qinlian decoction enhances the effect of PD-1 blockade in colorectal cancer with microsatellite stability by remodelling the gut microbiota and the tumour microenvironment. Cell Death Dis (2019) 10 415:43796. 10.1038/s41419-019-1638-6 31138779 PMC6538740

[94] Takaku S Shimizu M Takahashi H Japanese Kampo medicine ninjin’yoeito synergistically enhances tumor vaccine effects mediated by CD8(+) T cells. Oncol Lett (2017) 13:3471–8. 10.3892/ol.2017.5937 PMC5431575 28529575

[95] Lu SB Peng XF Effect of guipi decoction combined with NK cell on the quality of life and immune function in patients with advanced malignant neoplasm. Chin J Cancer Prev Treat (2018) 025:1733–6. 10.16073/j.cnki.cjcpt.2018.24.011

[96] Koga N Moriya F Waki K Yamada A Itoh K Noguchi M Immunological efficacy of herbal medicines in prostate cancer patients treated by personalized peptide vaccine. Cancer Sci (2017) 108:2326–32. 10.1111/cas.13397 PMC5715291 28898532

[97] Ishikawa S Ishikawa T Tezuka C Asano K Sunagawa M Hisamitsu T Efficacy of Juzentaihoto for Tumor Immunotherapy in B16 Melanoma Metastasis Model. Evid-Based Compl Alt Med (2017) 2017:6054706. 10.1155/2017/6054706 PMC5329671 28286532

[98] Lam W Ren Y Guan F Jiang Z Cheng W Xu CH Mechanism Based Quality Control (MBQC) of Herbal Products: A Case Study YIV-906 (PHY906). Front Pharmacol (2018) 9:1324. 10.3389/fphar.2018.01324 30510512 PMC6252377

[99] Liu S He X Man VH Ji B Wang J New application of in silico methods in identifying mechanisms of action and key components of anti-cancer herbal formulation YIV-906 (PHY906). Phys Chem Chem Phys (2019) 21:23501–13. 10.1039/C9CP03803E 31617551

[100] Teng Y Xiao JR Lin P Zhang J Effects of Combination of Aidi Injection and Neoadjuvant Chemotherapy on Patients with Non-Small-Cell Lung Cancer and Number of CD4~+CD25~+T Regulatory Cells. Chin J Exp Tradit Med Formulae (2012) 18:291–4. 10.13422/j.cnki.syfjx.2012.18.095

[101] Huang ZR Chen YM Lin H Lin JP Chen NJ Effect of Zilongjin Tablets on immune function of patients with lung cancer after operation. Chin Tradit Herbal Drugs (2019) 50:2941–4. 10.7501/j.issn.0253-2670.2019.12.029

[102] Wang H Li XJ Cai ZH Wang JH Xia YS Effect of Shenghe Powder on Recurrence and Metastasis Rate of Post-operative Triple-negative Breast Cancer. Chin Arch Tradit Chin Med (2018) 36:2983–6. 10.13193/j.issn.1673-7717.2018.12.043

[103] Zhu YL Yi FT The effect of γ knife the combined application of danshen injections on immune function in patients with primary liver cancer. Chin J Integrated Tradit Western Med Liver Dis (2020) 30:111–3. 10.3969/j.issn.1005-0264.2020.02.005

[104] Yuan TW Wen SW Dang ZJ Zhang XQ Chang JP Xue YQ Regulation of immune functions by combined Jinlong capsule and interventional therapy in patients with primary liver cancer. Chin J Clin Oncol (2013) 40:1116–8. 10.3969/j.issn.1000-8179.20130206

